# Rates and indications of caesarean section deliveries in Bhutan 2015–2019: a national review

**DOI:** 10.1186/s12884-021-04173-x

**Published:** 2021-10-18

**Authors:** Thinley Dorji, Phurb Dorji, Sonam Gyamtsho, Saran Tenzin Tamang, Tshering Wangden, Sangay Wangmo, Don Eliseo Lucero Prisno

**Affiliations:** 1 Jigme Dorji Wangchuck National Referral Hospital, Thimphu, 11001 Bhutan; 2 Kidu Mobile Medical Unit, His Majesty’s People’s Project, Thimphu, Bhutan; 3 Khesar Gyalpo University of Medical Sciences of Bhutan, Thimphu, Bhutan; 4 Central Regional Referral Hospital, Gelegphu, Bhutan; 5 London School of Hygiene and Tropical Medicine, London, UK

**Keywords:** Obstetric delivery, Maternal mortality, Neonatal mortality, Obstetric surgical procedure

## Abstract

**Background:**

Bhutan has made much efforts to provide timely access to health services during pregnancy and increase institutional deliveries. However, as specialist obstetric services became available in seven hospitals in the country, there has been a steady increase in the rates of caesarean deliveries. This article describes the national rates and indications of caesarean section deliveries in Bhutan.

**Methods:**

This is a review of hospital records and a qualitative analysis of peer-reviewed articles on caesarean deliveries in Bhutan. Data on the volume of all deliveries that happened in the country from 2015 to 2019 were retrieved from the Annual Health Bulletins published by the Ministry of Health. The volume of deliveries and caesarean deliveries were extracted from the Annual Report of the National Referral Hospital 2015–2019 and the data were collected from hospital records of six other obstetric centres. A national rate of caesarean section was calculated as a proportion out of the total institutional deliveries at all hospitals combined. At the hospital level, the proportion of caesarean deliveries are presented as a proportion out of total institutional deliveries conducted in that hospital.

**Results:**

For the period 2015–2019, the average national rate of caesarean section was 20.1% with a statistically significant increase from 18.1 to 21.5%. The average rate at the six obstetric centres was 29.9% with Phuentsholing Hospital (37.2%), Eastern Regional Referral Hospital (34.2%) and Samtse General Hospital (32.0%) reporting rates higher than that of the National Referral Hospital (28.1%). Except for the Eastern Regional Referral and Trashigang Hospitals, the other three centres showed significant increase in the proportion of caesarean deliveries during the study period. The proportion of emergency caesarean section at National Referral Hospital, Central Regional Referral Hospital and the Phuentsholing General Hospital was 58.8%. The National Referral Hospital (71.6%) and Phuentsholing General Hospital reported higher proportions of emergency caesarean sections (64.4%) while the Central Regional Referral Hospital reported higher proportions of elective sections (59.5%). The common indications were ‘past caesarean section’ (27.5%), foetal distress and non-reassuring cardiotocograph (14.3%), failed progress of labour (13.2%), cephalo-pelvic disproportion or shoulder dystocia (12.0%), and malpresentation including breech (8.8%).

**Conclusion:**

Bhutan’s caesarean section rates are high and on the rise despite a shortage of obstetricians. This trend may be counterproductive to Bhutan’s efforts towards 2030 Sustainable Development Goal agendas and calls for a review of obstetric standards and practices to reduce primary caesarean sections.

## Background

Caesarean section is a life-saving intervention for women and newborns that can effectively prevent maternal and perinatal mortality and morbidity [1, 2]. Low- and middle-income countries with poor maternal and neonatal indicators are recommended to increase access to caesarean section through the establishment of surgical centres and training of human resources [1]. There is a marked disparity in access to caesarean section surgeries across countries and within regions of the country [3]. While the global rates of children born via caesarean section has increased from 12.1% (16 million) in 2000 to 21.1% (29.7 million) in 2015, there are unmet needs for caesarean section where services are available to only 4.1% in Western and Central Africa and 6.2% in Eastern and Southern Africa [3]. On the other hand, Latin America and Caribbean region has very high rates of 44.3% followed by 32.0% in North America [4].

Bhutan is a small country with a population of 0.76 million situated in the eastern Himalayas that has made steady progress in maternal and child health indicators over the last three decades [5]. Within the framework of the Millennium Development Goals, Bhutan was among the nine countries that achieved the greatest relative reduction of maternal mortality ratio from 560 in 1990 to 86 per 100,000 live births in 2012 [6, 7]. The country also achieved reduction in infant mortality rate from 61 per 1000 live births in 2000 to 30 per 1000 live births in 2012 and a neonatal mortality rate of 21 per 1000 live births in 2012 [7]. The rate of stillbirths in 2015 among institutional deliveries, which covered 86% of all births in the country, was 10 per 1000 live births, much lower than the global estimate of 18.4 per 1000 live births [8, 9].

Much of these successes have resulted from the government’s consistent investment in maternal and child health programmes [5]. Bhutan started the Safe Motherhood Initiative in 1987 that promoted uptake of antenatal care, skilled-birth attendance or institutional delivery of babies and maternal and child immunizations [5]. While the government expanded its health network to include > 95% of its population within three-hours walking distance from a health centre, the government promoted institutional delivery as a key strategy towards improving maternal and child health outcomes. Under the Reproductive, Maternal and Child Health Programme, antenatal care is provided free of cost with government encouraging early booking visits and at least eight antenatal contacts during which the mothers are educated on obstetric danger signs and a birth preparedness plan encouraging institutional deliveries [10]. Bhutan has reported remarkable increase in institutional delivery coverage from 10% in 1994 to 19.8% in 2000, 73.7% in 2012 and 94.5% in 2020 [6, 7, 11, 12]. All antenatal, delivery and post-natal care of mother and child are provided through government hospitals, no private clinics exist in Bhutan [13]. The antenatal package through Bhutan Every Newborn Action Plan 2016–2023 promotes antenatal exercise and relaxation training to increase the uptake of normal delivery; at present, the service is only available at the National Referral Hospital [14]. The maternal exercise programme catered to 12,503 clients in 2018 and is a highly sought after programme [15].

As institutional deliveries increased, so has the percentage of deliveries conducted through caesarean section in Bhutan. In the South Asia region, as institutional deliveries increased from 29.6% in 2000 to 71% in 2015, the rates of caesarean section also increased from 7.2 to 18.1% in the same period [3]. Similarly, Bhutan has reported very high caesarean section rates: 32.4% in 2019 at the National Referral Hospital, Thimphu and 34.4% between 2016 and 2018 at the Eastern Regional Referral Hospital, Monggar [16, 17]. However, these figures are from only two of the seven obstetric centres in the country and may not be representative of the national caesarean section rate. This article reports the rates of caesarean section from all obstetrics centres in the country and its interpretation within the context of Bhutan’s effort to achieve the 2030 Sustainable Development Goals of reducing maternal mortality ratio to < 70 per 100,000 live birth and to reduce neonatal mortality to 12 per 1000 live births [18].

## Research method

### Study design

This was a descriptive study with a review of hospital records of deliveries (total and caesarean) between 2015 and 2019 with additional qualitative analysis of peer-reviewed articles related to caesarean deliveries in Bhutan.

### Setting

**Fig. 1:**
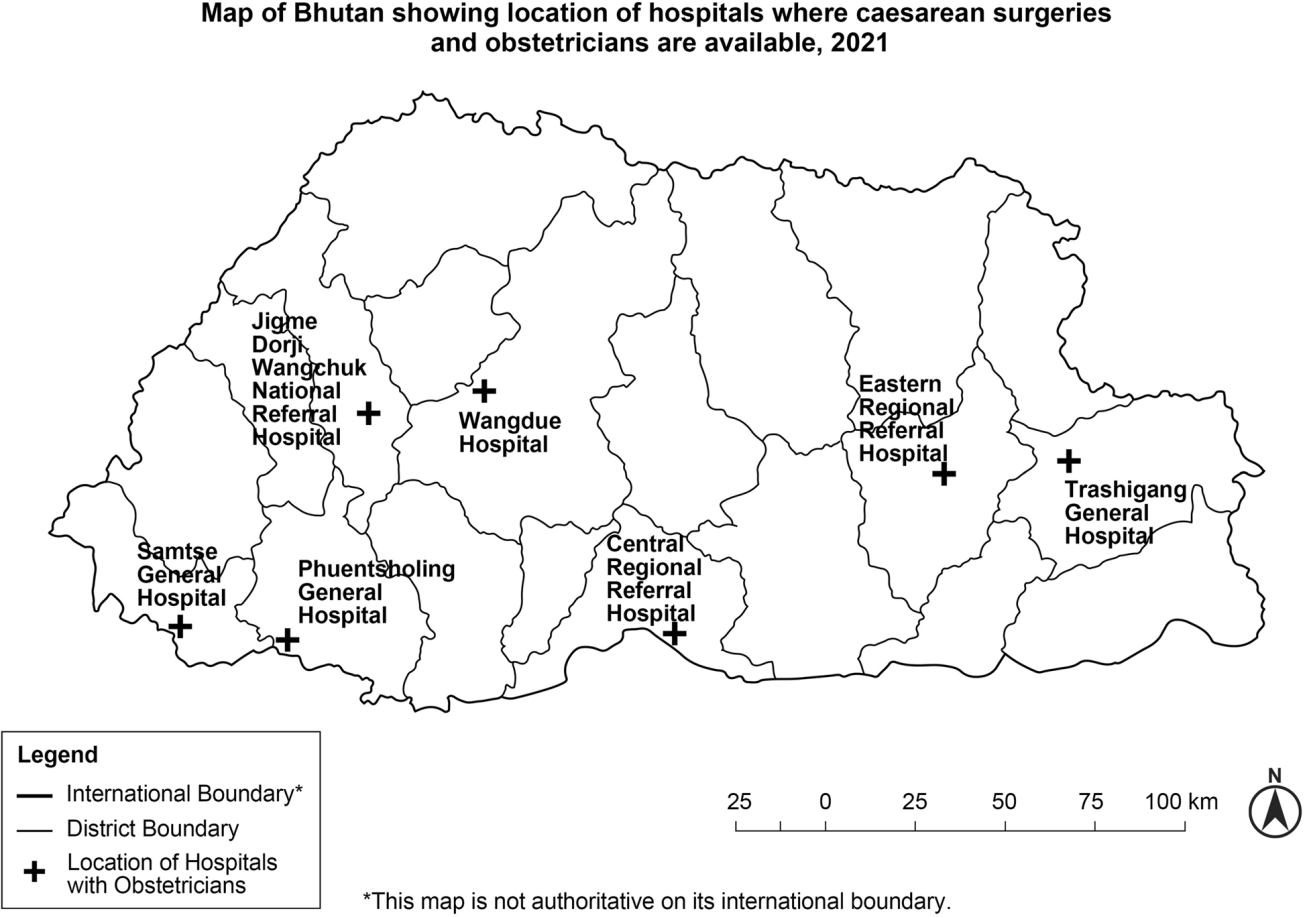
Map of Bhutan showing its 20 districts and the seven hospitals where caesarean section surgeries are available as of 2021*

During the 2017 census, Bhutan had 196,297 women in the reproductive age group (15–49 years) with a general fertility rate of 57.3 and total fertility rate of 1.7 per 1000 women [19]. In the 1990s and 2000s, Bhutan had very few obstetricians and gynaecologists. Much of skilled birth attendance were performed by Health Assistants, nurses and midwives [5, 20]. From 1994 to 2004, emergency obstetric care excluding caesarean section, were offered by doctors through the seven designated Emergency Obstetric Care Centres (EmOC) [5]. As more obstetrician-gynaecologists became available in the country, the services were provided through the referral hospitals and designated obstetric centres (Fig. 1). These centres are located across seven districts that provide strategic cover to the catchment population.

Bhutan has a three-tiered healthcare system with Primary Health Centres and 10-bedded hospitals at the primary level; district and general hospitals at the secondary level – four of which in Samtse, Phuentsholing, Trashigang and Wangdue Phodrang provide caesarean sections; and three referral hospitals at the tertiary level – Jigme Dorji Wangchuck (JDW) National Referral Hospital in Thimphu, Regional Referral Hospitals in Gelegphu and Monggar (Fig. 1). In 2020, Bhutan had 12 obstetrician-gynaecologists available in these seven strategic locations. Maternal and child health services such as antenatal care, immunization of mother and child, and normal deliveries are conducted at all levels of the hospitals under the National Reproductive, Maternal and Child Health Programme; Bhutan does not have a private health care system.

The JDW National Referral Hospital, Thimphu is the largest hospital in the country with 381 beds and catered to in-patient load of 17,921 and outpatient load of 521,478 patients in 2019 [16]. Its Department of Obstetrics and Gynaecology catered to 28,334 outpatient clients, 5910 in-patients, 975 patients at the maternal-foetal medicine unit, conducted 4522 deliveries, performed 1463 caesarean sections and 1447 gynaecological surgeries in 2019 [16]. The Central Regional Referral Hospital, Gelegphu has 150 beds and catered to 137,981 patients in 2019. The Eastern Regional Referral, Monggar has 150 beds and catered to 58,824 patients in 2019. Phuentsholing General Hospital, with 60 beds, is located near the border with India and catered to 71,256 patients in 2019. Samtse General Hospital has 40 beds and is also situated along the border with India and catered to 49,380 in 2019. Trashigang General Hospital has 40 beds and is located in the eastern part of the country and catered to 32,914 patients in 2019. The Wangdue General Hospital, Wangdue Phodrang moved into a newly constructed 60-bedded hospital complex with operating theatre facilities in 2019 and catered to 42,723 patients [12]. The locations of these obstetric centres are shown in Fig. 1.

### Data sources and variables

The Ministry of Health, Royal Government of Bhutan publishes the Annual Health Bulletin containing health-related data and indicators. Data on the total number of deliveries in the country for the period 2015 to 2019 were extracted from the Annual Health Bulletins of the respective years [21–25]. Data on the total number of deliveries and deliveries via caesarean section that happened at the respective hospitals were collected by the investigators from hospital records: Central Regional Referral Hospital, Eastern Regional Referral Hospital, Samtse General Hospital, Phuentsholing General Hospital and Trashigang General Hospital. Data from the JDW National Referral Hospital were extracted from its Annual Reports of the years 2015–2019 [15, 16, 26–28]. The data for caesarean deliveries in Samtse General Hospital in 2017, Trashigang General Hospital in 2019 and Wangdue General Hospital in 2019 were included in the calculation of overall national rates but were not included in the calculation of the rates for the hospital for that particular year because full-time obstetricians were not available for the entire year. With regard to the indications for caesarean deliveries, data from the JDW National Referral Hospital were extracted from its Annual Report 2016 and 2018 [15, 27], data from Eastern Regional Referral Hospital were extracted from Tamang et al. [17] and the data from Phuentsholing General Hospital were collected by the investigators.

### Data analysis

Data were extracted using Microsoft Excel and analysed using EpiData Analysis 2.2.2.132 (EpiData Association, Odense, Denmark). The national rate of caesarean section was calculated based on the total number of caesarean sections and the total institutional deliveries reported in the Annual Health Bulletins 2015–2019. The rates of caesarean section at the six obstetric centres were based on the total number of caesarean sections and the total number of institutional deliveries. The proportion of elective and emergency caesarean sections are presented for the JDW National Referral Hospital [15, 27], Central Regional Referral Hospital and the Phuentsholing General Hospital. For the purpose of this study, elective caesarean sections were those that were scheduled for surgery with no trial of labour while emergency caesarean sections were those unplanned surgeries that were undertaken for foetal or maternal wellbeing. Those with multiple gestations were considered as a single delivery. The common indications of caesarean section were synthesized based on data from the JDW National Referral Hospital reported in its Annual Reports of 2016 and 2018 [15, 27] and data from the Eastern Regional Referral Hospital reported by Tamang et al. [17]. The number of caesarean deliveries at the national level through the years 2015–2019 and the number at the six obstetric centres were compared using Chi-squared test. *P* values less than 0.05 were considered significant.

### Ethics considerations

Administrative approval was obtained from the Policy and Planning Division, Ministry of Health and site approvals from administrators of the seven hospitals. Ethics approval was obtained from the Research Ethics Board of Health, Ministry of Health.

## Results

### Data quality

The volume of institutional deliveries was reported in all Annual Health Bulletins. Only the JDW National Referral Hospital published annual reports; the first report for the year 2015 contained only the total number of deliveries and the number born via caesarean section [26]. The information on the type of caesarean section (emergency or elective) could be retrieved only for the JDW National Referral Hospital, Central Regional Referral Hospital and Phuentsholing General Hospital. The indications for caesarean section were available from only two hospitals: JDW National Referral Hospital in 2016 and 2018 [15, 27] and the Eastern Regional Referral Hospital in 2016–2018 [17]; data from the JDW National Referral Hospital for 2017 were omitted because of gross inconsistencies [28].

### Proportion of caesarean deliveries

**Fig. 2:**
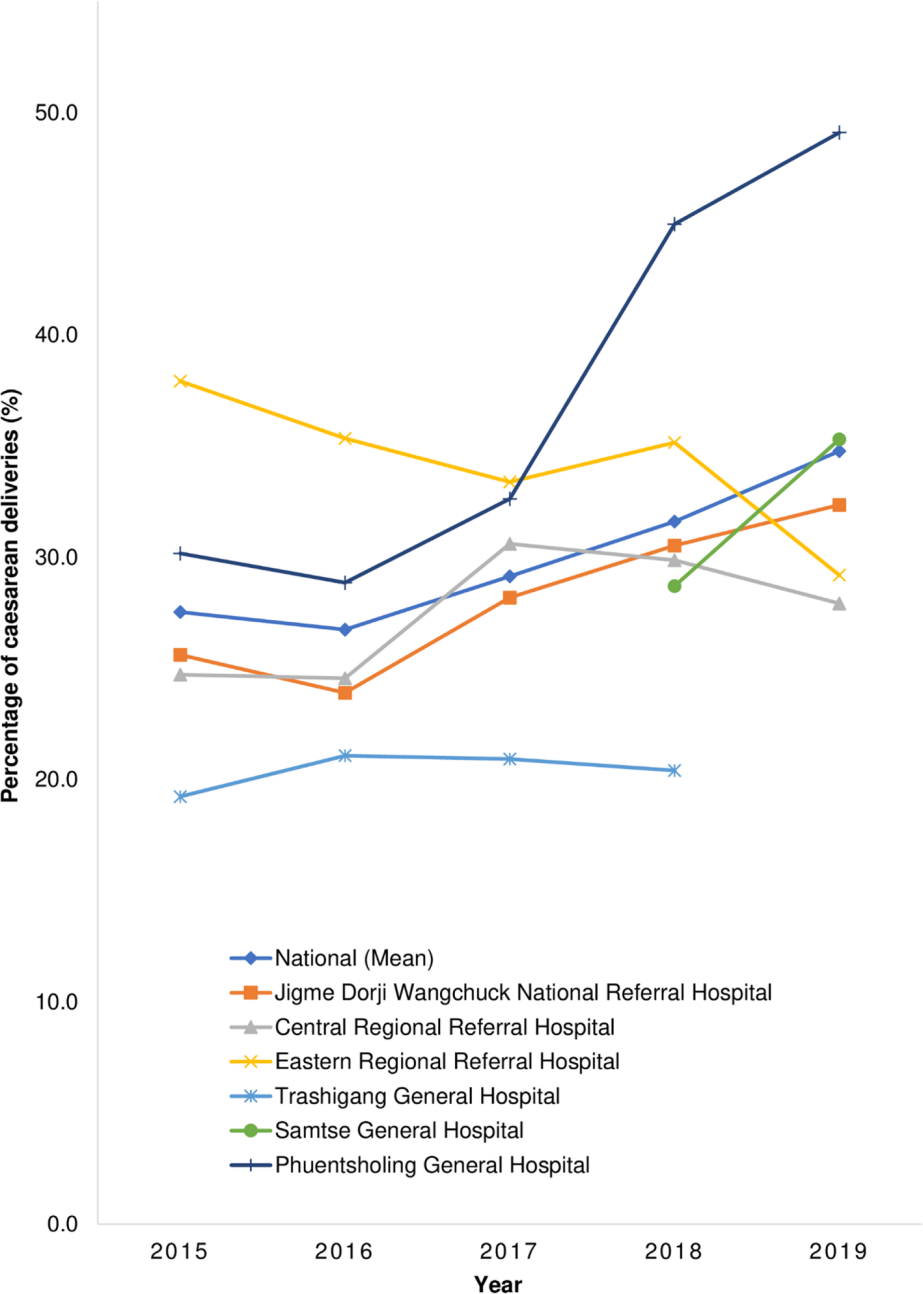
Trend of caesarean section deliveries in six of the seven obstetric centres in Bhutan, 2015–2019

During the period 2015–2019, there were 56,043 live births of which 54,324 (96.9%) were institutional deliveries and 10,919 were caesarean deliveries. The average national caesarean section rate for this period based on pooled data from seven obstetric centres was 20.1% showing statistically significant increasing trend from 18.1 to 21.5%, *p* < 0.001. The rate of caesarean deliveries at the six obstetric centres for the years when full-time obstetricians were in station was 29.9%, with the highest reported in Phuentsholing General Hospital (37.2%), followed by the Eastern Regional Referral Hospital (34.2%), Samtse General Hospital (32.0%), JDW National Referral Hospital (28.1%), Central Regional Referral Hospital (27.5%) and the lowest in Trashigang Hospital (20.4%). The JDW National Referral Hospital (*p* < 0.001), Central Regional Referral Hospital (*p* = 0.008), Phuentsholing General Hospital (*p* < 0.001) and Samtse General Hospital (*p* = 0.049) showed significant increase in caesarean section rates. The Eastern Regional Referral Hospital (*p* < 0.001) and Trashigang Hospital (*p* = 0.963) showed decrease in caesarean section rates. The details of volumes of deliveries including caesarean deliveries at the country-level are shown in Table 1 and the details at the hospital-level are shown in Table 2. The comparison of national- and hospital-level trends of proportions of caesarean deliveries is shown in Fig. 2.

**Table 1:** The proportion of caesarean deliveries at a national level in Bhutan, 2015–2019

Year	Total deliveries^**a**^	Facility deliveries		Total caesarean section deliveries		Chi-squared ***p*** value
		n	(%)	n	(%)^**b**^	
2015	10,873	10,291	(94.6)	1863	(18.1)	< 0.001
2016	11,430	10,902	(95.4)	1830	(16.8)	
2017	10,948	10,718	(97.9)	2143	(20.0)	
2018	11,134	10,980	(98.6)	2626	(23.9)	
2019	11,658	11,433	(98.1)	2457	(21.5)	
**Total**	56,043	54,324	(96.9)	10,919	**(20.1)**	

The total of caesarean deliveries are a sum of that reported at the Jigme Dorji Wangchuck National Referral Hospital, Thimphu; Central Regional Referral Hospital, Gelegphu; Eastern Regional Referral Hospital, Monggar; Samtse General Hospital, Samtse; Phuentsholing General Hospital, Chhukha; Trashigang General Hospital, Trashigang; and Wangdue General Hospital, Wangdue Phodrang

^a^The volume of deliveries based on the Annual Health Bulletin 2015–2019, Ministry of Health, Royal Government of Bhutan [21–25]. The volume of non-institutional deliveries can be calculated by subtracting volume of facility deliveries from the total deliveries

^b^National rates of caesarean delivery = Total caesarean section deliveries in the country / Total institutional or facility deliveries

**Table 2:** The proportion of caesarean deliveries among institutional deliveries at the seven obstetrics centres in Bhutan, 2015–2019

Obstetric centres	Year	Total deliveries	Normal delivery (%)^a^		Caesarean delivery (%)^b^			Chi-squared ***p*** value
			n	(%)	n	(%)	Average (%)	
Jigme Dorji Wangchuck National Referral Hospital, Thimphu^c^ [15, 16, 26–28]	2015	4035	–	–	1033	(25.6)	28.1	**< 0.001**
	2016	4523	3405	(75.3)	1081	(23.9)		
	2017	4553	3172	(69.7)	1283	(28.2)		
	2018	5081	3380	(66.5)	1551	(30.5)		
	2019	4522	2866	(63.4)	1463	(32.4)		
Central Regional Referral Hospital, Gelegphu	2015	793	597	(75.3)	196	(24.7)	27.5	**0.008**
	2016	782	590	(75.4)	192	(24.6)		
	2017	883	611	(69.2)	270	(30.6)		
	2018	941	660	(70.1)	281	(29.9)		
	2019	1021	736	(72.1)	285	(27.9)		
Eastern Regional Referral Hospital, Monggar	2015	836	499	(59.7)	317	(37.9)	34.2	**< 0.001**
	2016	682	419	(61.4)	241	(35.3)		
	2017	665	433	(65.1)	222	(33.4)		
	2018	987	633	(64.1)	347	(35.2)		
	2019	949	670	(70.6)	277	(29.2)		
Samtse General Hospital, Samtse	2017	274	263	–	11	–	–	–
	2018	425	303	(71.3)	122	(28.7)	32.0	**0.049**
	2019	351	227	(64.7)	124	(35.3)		
Phuentsholing General Hospital, Chhukha	2015	918	641	(69.8)	277	(30.2)	37.2	**0.001**
	2016	863	614	(71.1)	249	(28.9)		
	2017	898	605	(67.4)	293	(32.6)		
	2018	587	323	(55.0)	264	(45.0)		
	2019	553	281	(50.8)	272	(49.2)		
Trashigang Hospital, Trashigang	2015	208	166	(79.8)	40	(19.2)	20.4	0.963
	2016	318	249	(78.3)	67	(21.1)		
	2017	306	241	(78.8)	64	(20.9)		
	2018	299	238	(79.6)	61	(20.4)		
	2019	130	125	–	5	–	–	–
Wangdue General Hospital, Wangdue Phodrang^a^	2015	204	204	(100.0)	0	(0.0)	–	–
	2016	202	202	(100.0)	0	(0.0)		
	2017	251	251	(100.0)	0	(0.0)		
	2018	258	258	(100.0)	0	(0.0)		
	2019	263	232	–	31	–		

^a^The volume of non-institutional deliveries can be calculated by subtracting volume of facility deliveries from the total deliveries

^b^The data for caesarean deliveries in Samtse General Hospital in 2017, Trashigang General Hospital in 2019 and Wangdue General Hospital in 2019 were not included in the calculation of the rates for that particular year because obstetricians were not available for the entire duration of that year

^c^The volume of normal deliveries at Jigme Dorji Wangchuck National Referral Hospital, Thimphu in 2015 is not presented because the volume of instrumental deliveries were also a part of the data presented in the Annual Report 2015 [26]

### Types and indications of caesarean sections

Based on data from the JDW National Referral Hospital, Central Regional Referral Hospital and Phuentsholing General Hospital, the average proportion of emergency caesarean section was 58.8% and that of elective caesarean section was 41.0% (Table 3). The JDW National Referral Hospital (*p* < 0.001) and Phuentsholing General Hospital (*p* < 0.001) had higher proportions of emergency caesarean section. However, the Central Regional Referral Hospital reported the higher proportion of elective caesarean section at 59.5%, *p* < 0.001. The details of the proportions of emergency and elective caesarean sections are shown in Table 3.

**Table 3:** The proportions of emergency and elective caesarean sections among institutional deliveries at the Jigme Dorji Wangchuck National Referral Hospital, Central Regional Referral Hospital and Phuentsholing General Hospital, Bhutan, 2017–2019

Obstetric centre	Year	Total caesarean section	Emergency caesarean section		Elective caesarean section		Chi-squared ***p*** value
			%	Average (%)	%	Average (%)	
Jigme Dorji Wangchuck National Referral Hospital, Thimphu [15, 16, 28]	2017	1283^a^	72.3	71.6	26.3	28.1	**< 0.001**
	2018	1551	75.8		24.2		
	2019	1463	66.7		33.3		
Central Regional Referral Hospital, Gelegphu	2015	196	40.8	40.5	59.2	59.5	**< 0.001**
	2016	192	34.4		65.6		
	2017	270	33.3		66.7		
	2018	281	44.8		55.2		
	2019	285	49.1		50.9		
Phuentsholing General Hospital, Chhukha	2015	277	63.9	64.4	36.1	35.6	**< 0.001**
	2016	249	75.1		24.9		
	2017	293	70.6		29.4		
	2018	264	43.2		56.8		
	2019	272	69.1		30.9		

^a^Missing information for 17 cases

The most common indications of caesarean section (elective and emergency combined) at the JDW National Referral Hospital in 2016 and 2018, and the Eastern Regional Referral Hospital in 2016–2018 were ‘previous caesarean section’ (27.5%) followed by foetal distress and non-reassuring cardiotocograph (14.3%), failed progress of labour (13.2%), cephalo-pelvic disproportion or shoulder dystocia (12.0%), oligohydramnios (9.1%), malpresentation including breech presentation (8.8%), failed induction of labour (8.7%), foetal growth restriction (5.7%) and pre-eclampsia, eclampsia and hypertension (4.6%). At the Eastern Regional Referral Hospital Robson Group 5 (multi-parous, singleton, cephalic, full-term, with a previous caesarean section) was the most common indication (28.1%) of caesarean sections [17]. Table 4 shows the details of the indications of caesarean sections.

**Table 4:** The common indications of caesarean section among institutional deliveries at the Jigme Dorji Wangchuck National Referral Hospital, Thimphu and the Eastern Regional Referral Hospital, Bhutan, 2016–2018

Indications for caesarean section	Jigme Dorji Wangchuck National Referral Hospital^a^ [15, 27]		Eastern Regional Referral Hospital^a^ [17]			Average (%)
	2016 (%)	2018 (%)	2016 (%)	2017 (%)	2018 (%)	
Past caesarean section	17.5	30.6	29.4	28.7	31.2	27.5
Foetal distress & non-reactive cardiotocograph	25.2	16.6	10.1	12.1	7.3	14.3
Failed progress of labour	17.0	9.3	–			13.2
Cephalo-pelvic disproportion and shoulder dystocia	–	4.4	17.2	14.3	12.1	12.0
Oligohydramnios	–	–	9.1			9.1
Malpresentation including breech	8.9	8.9	8.5			8.8
Failed induction of labour	7.5	6.8	4.2	12.1	13.1	8.7
Foetal growth restriction	–	5.7	–			5.7
Pre-eclampsia, eclampsia and hypertension	–	3.5	5.7			4.6

^a^The hyphen represents data not present in these sources

## Discussion

### Proportion of caesarean deliveries

The rates of caesarean section deliveries reported during the period 2015–2019 at the national level are high and shows an increasing trend. The rates of three hospitals – Phuentsholing Hospital, Eastern Regional Referral Hospital and Samtse Hospital – were higher than that of the JDW National Referral Hospital. The high rates of caesarean section are a cause of concern with regard to the achievement of the 2030 Sustainable Development Agenda. Systematic reviews have shown that increase in caesarean section rates beyond 10–15% at a population level are no longer associated with reduced maternal or neonatal mortality rates as surgical risks outweigh benefits [4, 29]. High caesarean section rates are associated with increased risk of uterine rupture, abnormal presentation, ectopic pregnancy, still birth and preterm birth in a dose-dependent manner [2]. The risks also include altered immune development, increased likelihood of allergy, atopy, asthma and reduced gut microbiome diversity in the child [2]. While timely caesarean section leads to improved maternal and foetal outcomes, Bhutan’s high caesarean rates calls for a review of obstetric practices and childbirth practices in the country.

### Previous caesarean section

Previous caesarean section (Robson group 5) is a major contributor as women in this group undergo elective caesarean section in their subsequent pregnancies with no trial of Vaginal Birth After Caesarean (VBAC) [17]. A meta-analysis of 203 studies demonstrated that elective repeat caesarean section significantly increased maternal mortality compared with planned VBAC (1.34 versus 0.38 per 10,000), although VBAC was associated with significantly increased perinatal mortality compared with repeat caesarean section (13 versus 5 per 10,000) [30]. VBAC can be safely conducted if pregnant mothers are counselled at the time of antenatal visits and if proper assessment and birth preparedness plans are made [31]. In Bhutan, hospital policy changes are required to encourage VBAC among women with justifiable obstetric risks such as those with one previous caesarean section. Women and their partners must be invited for discussion on VBAC during their antenatal visits. In Bhutan, difficulty in continuous monitoring of women during labour, relative difficulty in accessing surgical facility and limited staff trained in VBAC are cited as challenges [17]. However, with the expanding human resource pool and improved infrastructure and facilities, there is a need to review the challenges and opportunities in initiating VBAC services in the country.

### Reducing primary caesarean section

In 2017, at the JDW National Referral Hospital, 282 out of 291 preterm deliveries (96.9%) were born by caesarean section – 143 (49.1%) via emergency caesarean and 139 (47.8%) via elective caesarean section [32]. In 2016, among 321 neonates with clinically diagnosed sepsis at the JDW National Referral Hospital, 92 (28.7%) were born by caesarean section; among 44 with culture-positive sepsis, 22 (50%) were born by caesarean section [33]. In 2017, among women at the three referral hospitals, 33 (30.3%) women among 109 with gestational diabetes and 146 (20.1%) among 617 women without gestational diabetes mellitus delivered by caesarean section, *p* = 0.367 [34]. A review of indications of caesarean sections in four selected centres (JDW National Referral Hospital, Eastern Regional Referral Hospital, Central Regional Referral Hospital and Samtse General Hospital) reported that compared to those women with two or more live children, women with no live child (adjusted odds ratio 2.26, 95% CI 1.23–4.14, *p* < 0.009) and those with only one live child (adjusted odds ratio 1.56, 95% CI 1.22–1.99, *p* < 0.001) had significantly higher odds of undergoing caesarean delivery [35].

In our review, the second major contributor of high caesarean section rates was malpresentations including breech presentations. While the majority of breech presentations were successfully delivered vaginally in the recent past, there is a noticeable decline in the availability of skilled personnel in the country who can conduct breech delivery. External cephalic version for term pregnancies with a trial of labour may be useful in settings where planned caesarean section are not easily available [36]. Although, infants born via vaginal breech had higher odds of brachial plexus injury, Apgar score < 7 at 5 min, intracranial haemorrhage or convulsions [37], planned caesarean section is not associated with a reduction of risk of death or neurodevelopmental delay in children at 2 years of age [38]. Though trial of labour among breech with high estimated foetal weight (≥ 3.8 kg) is likely to end up in caesarean section [39], the average weight of newborns is 3.2 (±0.4) kg in Bhutan suggesting lesser risk of caesarean section compared to elsewhere [37]. However, many countries in the region including Bhutan, practice 100% planned caesarean section for malpresentations. Given the current global practices and limited capacity for advanced neonatal care in the country, a careful review and a national policy on vaginal breech delivery is required.

Abnormal foetal heart rate tracing or suspected foetal distress is an emergency condition that warrants urgent caesarean section to prevent hypoxemia and acidaemia in the foetus. Foetal distress and non-reassuring cardiotocograph (CTG) contributed to a quarter of caesarean sections in 2016 at the JDW National Referral Hospital and it still remains an important contributor. CTG alone has high false positive rates in diagnosing foetal distress and requires adequate training of the interpreter. While CTG monitoring was not available in all centres that delivered babies in 2016 [40], an *iCTG* facility was introduced in 2020 to facilitate remote monitoring of CTG by obstetricians. The hospitals lack objective means to measure foetal distress such as foetal scalp electrode to assess foetal electrocardiograph and foetal blood gas sampling. Availability of more objective and accurate assessment can reduce false negative cases undergoing caesarean sections and reduce neonatal morbidity and mortality.

Foetal growth restriction contributed 5.7% and oligohydramnios contributed 9.1% of caesarean section. In Bhutan, the rate of pre-term delivery at the JDW National Referral Hospital in 2017 was 6.4% and the majority had low birth weight and were born via provider-initiated caesarean section [32, 33]. Among those delivered by caesarean section, the overall mortality was 11% and neonatal death due to sepsis was 8.1% [32, 33]. At a time when neonatal care services need to be strengthened in the country – capability of neonatal intensive care units to provide care for preterm babies, need of skilled human resources, expansion of intensive care services and availability of blood culture facilities – adequate antenatal and obstetric care standards should be met to reduce the proportion of preventable pregnancy complications leading to primary caesarean section. Therefore, the reduction of primary caesarean section remains an important intervention until the practice of elective caesarean for women with previous section and breech presentations are not reversed.

The rate of gestational diabetes in Bhutan in 2016 was 15% among 726 women in the three referral hospitals who underwent oral glucose tolerance test as per the study protocol with at least 8 h of fasting [34]. The rate of caesarean birth and the proportion of babies with macrosomia (birth weight > 4000 g) was higher among those women with gestational diabetes. The antenatal care package provided through the Reproductive, Maternal and Neonatal Health programme requires early booking, at least eight antenatal visits and timely ultrasound scans. However, a review of uptake of antenatal care in 2018 in the three referral hospital showed 67% of the women (584 out of 868) had late booking while 1% (13 women) gave birth with no antenatal visits [10]. If adequate antenatal follow up is achieved, deliveries can be planned and maternal and foetal complications detected early to plan appropriate delivery measures.

### Non-clinical interventions

The other drivers of caesarean section includes fear of labour pain, fear of pelvic floor damage and urinary incontinence, fear of negative effects on their sexuality or sexual relationships, and maternal requests [41]. In Bhutan, all deliveries are conducted in government health centres – there are no private clinics. Although deliveries are conducted free of cost, reported factors such as women in labour not being allowed to adopt preferred birth position, not allowing women to eat food and abuse from staff demonstrate lack of respectful maternity care [42]. While the government has initiated policies and procedures, additional efforts are urgently needed to create an environment for respectful normal deliveries in hospitals which may in turn reduce the proportion of maternal requests for elective caesarean sections.

The World Health Organization recommendations on non-clinical interventions to reduce unnecessary caesarean section include childbirth training workshops, nurse-led applied relaxation training programme, psychosocial couple-based programme and psycho-education to cope with fear of pain [43]. For organization-led initiatives to reduce the rates of caesarean section, the World Health Organization recommends improved adherence to evidence-based clinical practice, second-opinion policy on indications of caesarean sections and feedback and peer-review of caesarean indications [43]. The National Referral Hospital follows a standard operating procedure that brings consensus in practice and upholds the standards of care. However, such detailed guideline on the management of childbirth care and involvement of partner and family members in decision making is lacking in the hospitals. There is no national policy on audit and review of rates and indications of caesarean section in the country. Except for one study that has adopted the Robson classification to audit caesarean deliveries at the Eastern Regional Referral Hospital [17], the Reproductive, Maternal and Neonatal Health Programme does not mandate reporting using this classification system. In addition, Maternal Death Reviews, social- and professional pressure on events of still births, and maternal and neonatal deaths reflected as a performance indicator for individual obstetricians and hospitals allow for a low threshold for caesarean surgeries [5].

The World Health Organization also recommends a model of staffing based on care provided primarily by midwives with 24-h back up from an obstetrician who provides in-house labour and delivery coverage [43]. As of 2021, there were only 12 obstetricians available in the obstetric centres in the country with another seven under training at the Khesar Gyalpo University of Medical Sciences of Bhutan, the only medical university in the country. Obstetric duty coverage in all other hospitals is provided by medical officer with MBBS degrees. With an aim to reduce maternal and neonatal mortality, all pregnancies with suspected high risk are referred to these centres where specialist services are available. The government’s human resource projection should take into account future standards of obstetric and neonatal care, population growth, respectful maternity care during delivery and cater to psycho-social needs of women related to childbirth experiences.

### Limitations

This study reports rates of caesarean births from centres where pregnancies with high-risk are referred for specialist care. It may be by virtue of complicated nature of pregnancy that caesarean sections were warranted. However, with increased social mobility, easy access to transportation and a weak referral system that is easily bypassed, many women with normal pregnancies present for deliveries at these hospitals exposing to greater chances of having caesarean birth.

The higher rates of caesarean section at the Phuentsholing General Hospital, Eastern Regional Referral Hospital and Samtse General Hospital may be because of receiving higher volumes of referral of complicated pregnancies given the large base population served by these hospitals. The referred cases reach the obstetric centres often with delays and in urgencies requiring emergency caesarean sections. Therefore, this may not be a true representation of lower threshold for caesarean section and further studies are required to assess the rates among women who were referred versus those who present primarily to those hospitals.

## Conclusion

Bhutan has a high rate of caesarean section that are primarily driven by previous-caesarean section, non-reassuring cardiotocograph, failed progress of labour and malpresentations including breech. The country lacks a national guideline on obstetric standards and allows individual-driven practices that has low threshold for caesarean section. For Bhutan to achieve its 2030 Sustainable Development Goal agenda related to maternal and neonatal death, the present high rates of caesarean may be counterproductive and warrants an urgent need to review current practices in obstetrics.

## Data Availability

The datasets used and/or analysed during the current study available from the corresponding author on reasonable request.
